# *FLOWERING LOCUS T* indel variants confer vernalization-independent and photoperiod-insensitive flowering of yellow lupin (*Lupinus luteus* L.)

**DOI:** 10.1093/hr/uhac180

**Published:** 2022-08-24

**Authors:** Piotr Plewiński, Sandra Rychel-Bielska, Bartosz Kozak, Iván J Maureira-Butler, Muhammad Munir Iqbal, Matthew N Nelson, Michał Książkiewicz

**Affiliations:** Department of Gene Structure and Function, Institute of Plant Genetics, Polish Academy of Sciences, Poznań, Poland; Department of Genetics, Plant Breeding and Seed Production, Wroclaw University of Environmental and Life Sciences, Wrocław, Poland; Department of Genetics, Plant Breeding and Seed Production, Wroclaw University of Environmental and Life Sciences, Wrocław, Poland; Instituto de Producción y Sanidad Vegetal, Facultad de Ciencias Agrarias y Alimentarias, Universidad Austral de Chile, Valdivia, Chile; Centre for Plant Genetics and Breeding, The University of Western Australia, Perth, 6009, WA, Australia; Genomics WA, Joint initiative of Telethon Kids Institute, Harry Perkins Institute of Medical Research and The University of Western Australia, QEII campus, Nedlands, 6009, Western Australia, Australia; The UWA Institute of Agriculture, The University of Western Australia, Perth, Australia; Department of Gene Structure and Function, Institute of Plant Genetics, Polish Academy of Sciences, Poznań, Poland

## Abstract

Ongoing climate change has considerably reduced the seasonal window for crop vernalization, concurrently expanding cultivation area into northern latitudes with long-day photoperiod. To address these changes, cool season legume breeders need to understand molecular control of vernalization and photoperiod. A key floral transition gene integrating signals from these pathways is the *Flowering locus T* (*FT*). Here, a recently domesticated grain legume, yellow lupin (*Lupinus luteus* L.), was explored for potential involvement of *FT* homologues in abolition of vernalization and photoperiod requirements. Two *FTa* (*LlutFTa1a* and *LlutFTa1b*) and *FTc* (*LlutFTc1* and *LlutFTc2*) homologues were identified and sequenced for two contrasting parents of a reference recombinant inbred line (RIL) population, an early-flowering cultivar Wodjil and a late-flowering wild-type P28213. Large deletions were detected in the 5′ promoter regions of three *FT* homologues. Quantitative trait loci were identified for flowering time and vernalization response in the RIL population and in a diverse panel of wild and domesticated accessions. A 2227 bp deletion found in the *LlutFTc1* promoter was linked with early phenology and vernalization independence, whereas *LlutFTa1a* and *LlutFTc2* indels with photoperiod responsiveness. Comparative mapping highlighted convergence of *FTc1* indel evolution in two Old World lupin species, addressing both artificial selection during domestication and natural adaptation to short season environmental conditions. We concluded that rapid flowering in yellow lupin is associated with the de-repression of the *LlutFTc1* homologue from the juvenile phase, putatively due to the elimination of all binding sites in the promoter region for the AGAMOUS-like 15 transcription factor.

## Introduction

Synchronization of flowering time with a particular season is essential for the reproductive success of plants growing in climates experiencing significant annual cycles. Knowledge of the regulatory network underlying flowering control would facilitate the breeding of new plant varieties that are better adapted to target agroecosystems. This issue is becoming more urgent in the era of climate change consecutively narrowing the time window for spring sowing of vernalization-responsive crops [1, 2]. It is due to the reduction of the lengths of winter and spring in the Northern Hemisphere mid-latitudes, where temperate climate crops are cultivated [3]. As legume species have generally low tolerance to freezing temperatures [4], in colder regions of temperate climate, that include also major European cultivation areas, vernalization-responsive species are sown in early spring rather than in autumn to fulfil vernalization requirements without an excessive risk offrost damage. Nevertheless, higher temperature in winter resulting from climate change may also lead to incomplete fulfillment of vernalization requirement in some winter plant species,resulting in delayed flowering or a failure of floral induction [5].Indeed, a long-term study based on 47-year record (1954–2000) ofphenological changes revealed a progression of delayed flowering in natural populations with high vernalization requirements [1],whereas analysis of inter-annual sensitivity of winter wheat yields to vernalization degree days during 1975–2009 revealed its potential vulnerability to warming-mediated vernalization variationsin temperate climate [6]. Moreover, spring heat waves, that may occur more frequently in warmer climates, can erase epigeneticmarks of vernalization, resulting in de-vernalization and delayedflowering [7, 8].

Genetic and molecular regulation of flowering induction is best known in the model plant, *Arabidopsis thaliana* (L.) Heynh. Flowering induction pathways address both environmental factors such as vernalization, high temperature, photoperiod and light quality as well as endogenous signalling, such as the gibberellin pathway, ageing and carbohydrates [9]. These pathways converge in the transcriptomic regulation of floral integrator genes. The key floral pathway integrator responding to environmental signals (low and high temperature, photoperiod and light quality) is the *FLOWERING LOCUS T* (*FT*) gene [10, 11]. *Arabidopsis* has only two *FT*-like genes (*FT* and a one close homolog, *TWIN SISTER OF FT*), whereas legume genomes usually encode higher number of *FT*-like genes, assigned into three subclades, *FTa*, *FTb* and *FTc* [12, 13]. While *FT* retains this basic role in all flowering plants studied to date, it is unclear how many homologues perform the role of floral integrator in legumes. Moreover, involvement of particular *FT* homologues in photoperiod or vernalization response considerably vary between legume species [12, 14–18]. In the present study, we selected yellow lupin (*L. luteus* L.), an annual legume with wild winter annual and domesticated spring annual forms, as a model to explore the sequence and functional divergence of *FT* homologues.

Yellow lupin (*L. luteus* L.) is a grain legume crop natively distributed primarily across the coastal region of the Iberian Peninsula [19]. Yellow lupin evolved numerous adaptations to deal with the dry-summer Mediterranean climate (such as drought avoidance by early phenology) and biotic pressures (i.e. anthracnose and aphid resistance) occurring in this environment [20–23]. Yellow lupin has a very short history of domestication as compared to other legumes, because all the key milestones in converting the wild types to domesticated forms were achieved in the 20^th^ century [24]. Modern yellow lupin cultivars have low alkaloid and high protein content in seeds, and as such are currently considered as a highly nutritional alternative to soybean meal in animal diets [25]. Yellow lupin phenology studies revealed high variability of the vegetative period resulting mainly from differences in vernalization requirements and long photoperiod preferences [26, 27]. Depending on the application (green manure, biomass, or grain production) as well as climatic constraints (spring or winter sowing, the need of drought escape), breeding pressure on vernalization requirements and early phenology is different [28]. As the juvenile photoperiod-nonresponsive phase is relatively short in lupins [29], matching of periods with conditions ensuring fulfillment of vernalization and photoperiod requirements becomes challenging due to climate change. Determining the molecular components underlying existing variability in vernalization response and phenology would help to address this issue, enabling molecular-assisted breeding. Moreover, such a knowledge could facilitate studies in other vernalization-responsive legumes. Indeed, vernalization control in this large plant family differ from the *Arabidopsis* model because the majority of legumes do not have a key gene from the vernalization pathway, *FLOWERING LOCUS C* (*FLC*), whereas soybean, that has retained one such homologue, is vernalization independent because it evolved in a warm sub-tropical environment [30]. Early flowering based on thermoneutrality (vernalization independence) is the key agronomic trait of lupins, enabling their successful cultivation in temperate climates (spring-sown in Northern Europe or autumn sown in the mild Mediterranean-like climates of Australia) [29]. Quantitative trait loci (QTL) mapping revealed that flowering time in yellow lupin is controlled by several QTLs, with one major locus for vernalization response [31]. In the closely related species, narrow-leafed lupin, vernalization insensitivity is also based on a single locus, conferred by natural mutations that occurred during domestication period (*Ku* and *Jul*) [32, 33]. These mutations constitute two overlapping deletion variants in the promoter region of one of the *FT* homologues, *LanFTc1* gene [18, 34]. Recent yellow lupin mapping studies revealed collinearity between the linkage group carrying the major QTL for vernalization response and the narrow-leafed lupin genome scaffold carrying *LanFTc1* sequence [35, 36]. Flowering time in another related species, white lupin, was found to be under quantitative control, with two QTLs associated with *FTa* and *FTc* gene-based markers [37–40]. All these findings improved the knowledge on the key role of *FT* in flowering time control in legumes and driven our attention to the *FT* clade present in the yellow lupin genome.

In this study, the involvement of *FT* genes in flowering time control in yellow lupin was analyzed by several complementary approaches, including linkage and QTL mapping, gene sequencing and quantitative expression profiling under two contrasting photoperiod and vernalization conditions. Moreover, a yellow lupin germplasm diversity panel carrying wild and domesticated accessions was phenotyped for selected phenology traits and vernalization responsiveness in controlled conditions as well as genotyped for the presence of indel polymorphisms using PCR-based markers spanning the whole *FT* gene sequences and promoter regions. The study provided several independent lines of evidence to support the involvement of three *FT* homologs in flowering time control in yellow lupin, with the *LlutFTc1* homologue playing the key role in domestication driven by reduction of juvenile phase and elimination of vernalization requirements to induce flowering.

## Results

### Yellow lupin phenology is strongly determined by genotype

Phenotypic data of phenology traits analyzed with and without pre-sowing vernalization under ambient long day photoperiod were obtained for 109 yellow lupin accessions in the 2016 trial and for 111 accessions in the 2017 and 2019 trials (Tables S1 and S2). High variability between particular accessions in phenology in the absence of vernalization was observed, ranging from 42.5 ± 2.2 (PRH444/14) to 82.4 ± 5.5 (Biscainhos-4) days to the first floral bud emergence, from 50.7 ± 1.4 (PRH444/14) to 91.8 ± 5.3 (Biscainhos-4) days to the onset of flowering and from 68.3 ± 2.7 (Idol) to 101.3 ± 4.7 (Biscainhos-4) days to the end of flowering on the main stem. Accessions in the diversity panel differed also in vernalization requirements, ranging from full thermoneutrality to very high responsiveness, manifested by acceleration of transition from vegetative to generative growth phases by 3 weeks. Estimated marginal mean pairwise comparisons revealed that 22 lines were fully thermoneutral for bud emergence, 29 for start of flowering, 18 for end of flowering, and 9 lines for all of these traits (Table S3). The broad sense heritability coefficients of phenology traits in the yellow lupin diversity panel were high in the absence of vernalization, ranging from 74.9% to 81.4% and moderately high with vernalization treatment, ranging from 55.6% to 58.9% (Table 1).

**Table 1:** Genetic parameters calculated by linear mixed-effect model for selected phenology traits observed in a diversity panel of 111 yellow lupin accessions in long day conditions

**Parameters**	**BE** ^**1**^	**BE + v**	**SF**	**SF + v**	**EF**	**EF + v**
Phenotypic variance	97.1	75.5	93.3	69.1	69.0	51.5
Broad-sense heritability (%)	78.8	55.6	81.4	58.5	75.0	58.9
Heritability on the mean basis (%)	94.7	83.8	95.7	85.9	92.2	84.3
Selective accuracy (%)	0.97	0.92	0.98	0.93	0.96	0.92
Genotype-environment correlation	0.58	0.70	0.54	0.66	0.73	0.78
Genotypic coefficient of variation	14.25	11.93	12.71	10.24	8.85	7.24
Residual coefficient of variation	4.80	5.85	4.10	4.99	2.66	2.83

Phenotypic trait abbreviations are as follows: the number days to floral bud emergence without vernalization (BE) and with vernalization (BE+v), the number of days to start of flowering without vernalization (SF) and with vernalization (SF + v), and the number of days to end of flowering without vernalization (EF) and with vernalization (EF + v).

### Large indels series are present in the promoter regions of all yellow lupin *FT* homologs

**Figure 1:**
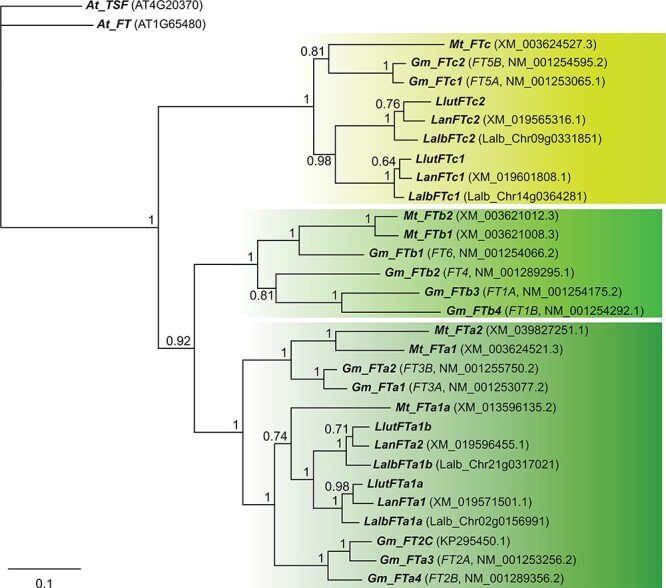
Majority rule consensus of 12 502 trees found in a Bayesian analysis of selected legume *FLOWERING LOCUS T* (*FT*) genes. Numbers are posterior probabilities. The length of coding sequence alignment was 537 nucleotides. Abbreviated species names are provided as follows: *At*, *Arabidopsis thaliana*; *Gm*, *Glycine max*; *Lan*, *Lupinus angustifolius*; *Mt*, *Medicago truncatula*; *Llut*, *Lupinus luteus*; *Lalb*, *Lupinus albus*. *L. luteus* genes were identified in this study, *L. albus* sequences were extracted from the reference genome [42], whereas the remaining sequences were derived from the recent phylogenetic studies [12, 14, 41]. NCBI accession numbers or *L. albus* gene names [42] were provided in parentheses.

BLAST analysis of yellow lupin draft genome assembly using *L. angustifolius FT* genes identified four *FT* homologues. Aligning these homologues with other legume *FT* homologues allowed the assignment of these homologues (by Bayesian inference) to the *FTa* clade (*LlutFTa1a* and *LlutFTa1b*) and the *FTc* (*LlutFTc1* and *LlutFTc2*) clade (Fig. 1). This analysis confirmed also reported lineage-specific duplications of *FTa* and *FTc* homologs in lupins [41].

Four yellow lupin accessions differing in domestication status and phenology were selected for further studies: PRH444/14 (Polish breeding line, very early flowering and thermoneutral), Wodjil (Australian cultivar, early flowering and near-thermoneutral), Parys (Polish cultivar, intermediate flowering and responsive to vernalization), and P28213 (Azorean wild population, late flowering and highly responsive to vernalization). Phenotyping in climatic chambers under two contrasting photoperiods confirmed variability in earliness and vernalization responsiveness observed between these accessions in greenhouse (Table 2).

**Table 2:** Number of days from sowing to the first bud, flower and pod in four *L. luteus* lines selected for sequencing of *FT* genes

**Line**	**Vernalization**	**Days to first bud**	**Days to first flower**	**Days to first pod**
**8-hour photoperiod**				
P28213	−	DNF^1^	DNF	DNF
	+	60.3 ± 4.9^2^	70.5 ± 5.2	78.4 ± 3.1
Parys	−	67.0 ± 1.2	74.1 ± 1.6	84.3 ± 1.5
	+	48.6 ± 3.3	54.6 ± 3.6	64.2 ± 2.8
PRH444	−	40.6 ± 2.9	47.4 ± 3.1	56.1 ± 3.5
	+	36.6 ± 8.1	42.1 ± 7.9	52.1 ± 9.1
Wodjil	−	46.6 ± 0.5	54.2 ± 1.1	60.6 ± 0.7
	+	40.7 ± 0.5	47.4 ± 0.5	52.3 ± 0.7
**16-hour photoperiod**				
P28213	−	73.0 ± 4.6	78.5 ± 4.2	86.5 ± 4.2
	+	53.8 ± 1.3	60.8 ± 1.9	67.5 ± 1.5
Parys	−	46.2 ± 6.3	54.3 ± 6.0	63.1 ± 6.0
	+	40.7 ± 3.2	48.2 ± 3.4	57.0 ± 3.1
PRH444	−	30.4 ± 2.4	37.7 ± 2.4	43.0 ± 2.3
	+	31.2 ± 1.1	37.6 ± 1.5	43.4 ± 2.3
Wodjil	−	40.5 ± 2.1	48.0 ± 2.1	54.3 ± 2.2
	+	34.0 ± 0.0	40.7 ± 1.3	48.6 ± 1.2

1 plants did not flower.

2 standard deviation.

**Figure 2:**
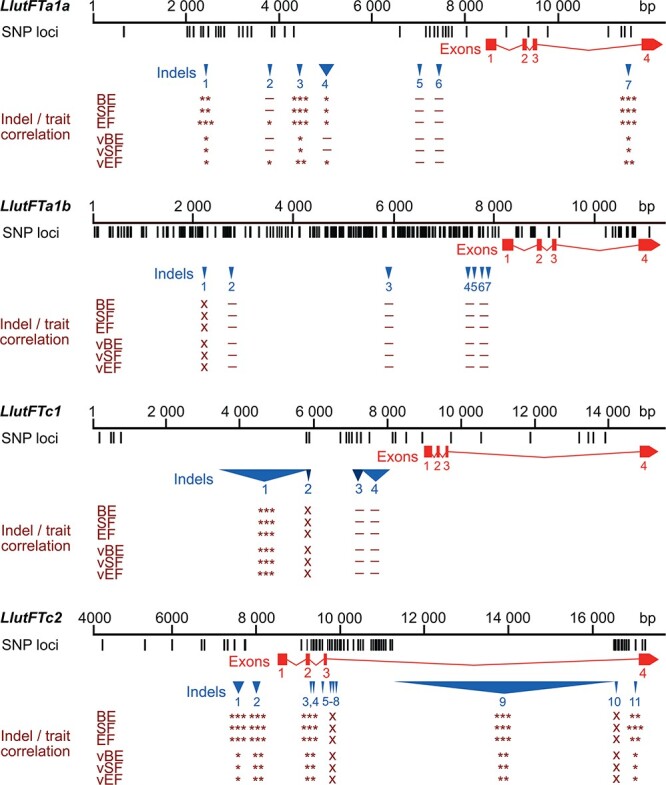
Sequence polymorphism revealed in the *Lupinus luteus FLOWERING LOCUS T* (*LlutFTa1a*, *LlutFTa1b*, *LlutFTc1* and *LlutFTc2*) genes. Black tags visualize SNP and short (≤5 bp) indel loci whereas red rectangles and blue triangles show exons and large (≥6 bp) indels, respectively. *P*-value of Spearman’s rank correlation coefficient, calculated for the phenology traits (BE, time to floral bud emergence; SF, time to start of flowering; and EF, time to end of flowering) and for the vernalization responsiveness of these traits (vBE, vSF and vEF, respectively), was shown in the following scheme: ^***^, *p* < 0.0001; ^**^, 0.0001 ≤ *p* < 0.001; ^*^, 0.001 ≤ *p* ≤ 0.05; −, *p* > 0.05 (not significant); x, not analyzed.

Full *FT* sequences, including ~8 kbp promoter regions, were retrieved by sequencing and assembly of overlapping PCR products (Table S4). This revealed the presence of indels in the promoter and other regions. Then, PCR-based screening of the diversity panel was performed. From the 76 primer pairs tested, 21 revealed indel polymorphism detectable under the resolution of 2% agarose gel electrophoresis with the minor allele frequency ranging from 0.009 to 0.234 (Table S5). Eight different long (≥6 bp) indel variants were identified for the *LlutFTa1a* gene, seven for *LlutFTa1b*, four for *LlutFTc1*, and eleven for *LlutFTc2* (Table 3, Fig. 2).

**Table 3:** Major indels (length ≥ 6 bp) identified in *FT* genes in a diversity panel of 111 yellow lupin accessions

**Gene**	**Indelno.**	**Region**	**Locus(bp)** ^1^	**Length(bp)**
*LlutFTa1a*	1	promoter	2401	12
	2	“	3802	12
	3	“	4418	58
	4	“	4868	245
	5	“	7071	15
	6	“	7407	59
	7	intron3	11 501	11
	8	5’UTR	12 370	6
*LlutFTa1b*	1	promoter	2219	24
	2	“	2752	8
	3	“	5857	44
	4	“	7478	6
	5	“	7576	46
	6	“	7724	10
	7	“	7787	73
*LlutFTc1*	1	promoter	3590	2227
	2	“	5817	21
	3	“	7177	305
	4	“	7325	687
*LlutFTc2*	1	promoter	7403	128
	2	“	7921	96
	3	intron2	9352	46
	4	“	9398	14
	5	“	9575	7
	6	intron3	9710	7
	7	“	9717	14
	8	“	9737	6
	9	“	11 247	5269
	10	“	16 541	9
	11	“	17 039	32

1 Position in the sequence alignment (see Supplementary File 1)

Sequencing performed for PRH444/14, Wodjil, Parys and P28213 accessions revealed, besides long indels, numerous SNPs and/or short (≤ 5 bp) indels, namely 48 in *LlutFTa1a*, 258 in *LlutFTa1b* (including one in the fourth exon), 32 in *LlutFTc1* and 81 in *LlutFTc2* (including one in the second exon and two in the fourth exon). In *LlutFTa1a*, *LlutFTa1b* and *LlutFTc1*, the majority of SNPs were localized in the promoters, accounting for 41, 213, and 24 SNPs, respectively. In *LlutFTc2*, the majority of SNPs (56) were found in the third intron. FGENESH+ provided identical protein sequence predictions for all nucleotide variants. The list of polymorphic loci is presented in Table S6 whereas FASTA alignments in Supplementary File 1.

### *LlutFTa1a, LlutFTc1* and *LlutFTc2* indels are strongly associated with flowering time and vernalization responsiveness

Three indel markers from the *LlutFTa1a*, one from the *LlutFTc1* and six from the *LlutFTc2* revealed statistically significant correlation with all phenology traits observed in a 3-year series of greenhouse experiments, as well as with vernalization responsiveness (understood as a shift in the BLUP for number of days in vernalized variant versus non-vernalized) (Table S7, Fig. 2). Thus, a large indel from the *LlutFTc1* promoter (2227 bp, indel 1) showed the highest association with phenology traits (*Spearman’s rank correlation coefficient, rho[ρ]**-**value, from* 0.70 to 0.72) and with vernalization responsiveness (*ρ-value* from −0.66 to 0.53) among all analyzed markers. Interestingly, this *LlutFTc1* indel allele was found only in domesticated germplasm except one landrace originating from Palestine (Palestyna-5). It should be noted that two other *LlutFTc1* promoter indel markers (indels 3 and 4) did not reveal significant correlation with any of the phenology traits.

**Figure 3:**
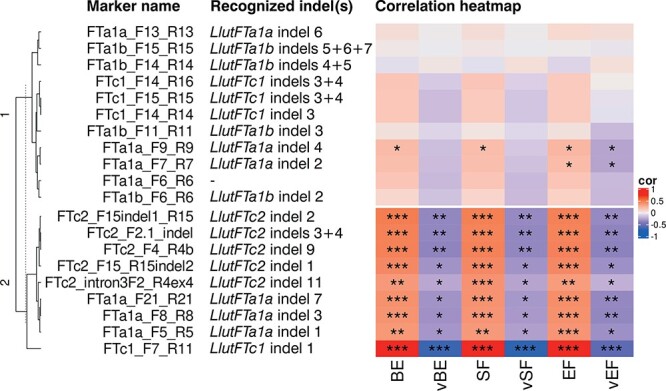
Heatmap showing marker clustering and Spearman’s rank correlation coefficients calculated for the diversity panel of 111 yellow lupin accessions using allelic diversity of *LlutFTa1a*, *LlutFTa1b*, *LlutFTc1* and *LlutFTc2* gene-based markers. Phenotypic trait abbreviations are as follows: BE, the number days to floral bud emergence (without vernalization); vBE, influence of vernalization on BE; SF, the number of days to start of flowering; vSF, influence of vernalization on SF; EF, the number of days to end of flowering; vEF, influence of vernalization on EF. Asterisk (^*^) indicates significant correlations in the following scheme: ^***^, *p* < 0.0001; ^**^, 0.0001 ≤ *p* < 0.001; ^*^, 0.001 ≤ *p* ≤ 0.05.

Five *LlutFTc2* indels, namely indels 1 and 2 from the promoter, indels 3 and 4 from the second intron and indel 9 from the third intron (carrying a large *Copia*-like retrotransposon insertion) also revealed significant correlation with phenology traits, but considerably lower than the large *LlutFTc1* promoter indel 1 (*ρ-value* from 0.43 to 0.48) as well as moderate correlation with vernalization response (*ρ-value* from −0.33 to −0.28). The most 3’ *LlutFTc2* indel 11, localized close to the fourth exon, showed significant but moderate correlation with phenology (*ρ-value* from 0.35 to 0.39) and vernalization responsiveness (*ρ-value* from −0.19 to −0.23). Three *LlutFTa1* indels (indels 1 and 3 from the promoter and indel 7 from the third intron) had similar correlation with vernalization response as four *LlutFTc2* indels (*ρ-value* from −0.33 to −0.25) and lower correlation with phenology (*ρ-value* from 0.35 to 0.39). Another two *LlutFTa1a* promoter indels (2 and 4) revealed significant correlation with one or three traits (*ρ-value* from 0.20 to 0.22), and vernalization response for the end of flowering (*ρ-value* − 0.22 and − 0.23). Two rare *LlutFTa1* alleles (FTa1a_F6_R6 presence/absence and indel 6) and all studied *LlutFTa1b* indels (2–7) did not reveal significant correlation with any trait. Marker grouping based on the distribution of *FT* indel polymorphism in analyzed yellow lupin lines revealed the presence of two major clusters, one carrying markers which revealed statistically significant correlation with all observed phenotypic traits and the other composed of the remaining markers (Fig. 3).

**Figure 4:**
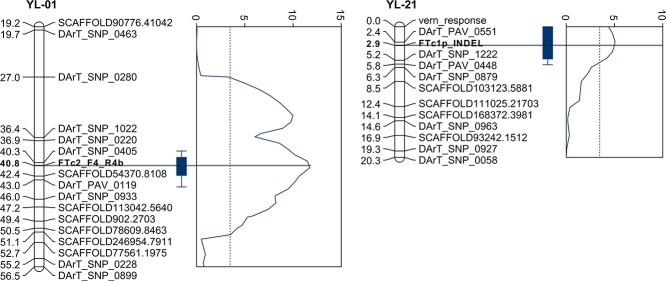
Two linkage groups (YL-01 and YL-21) from the yellow lupin linkage map carrying newly developed *LlutFTc1* and *LlutFTc2* markers and major quantitative trait loci for flowering time observed in a recombinant inbred line mapping population. Boxes indicate LOD_max_-1 intervals, whereas whiskers extends to LOD_max_-2 intervals.

**Figure 5:**
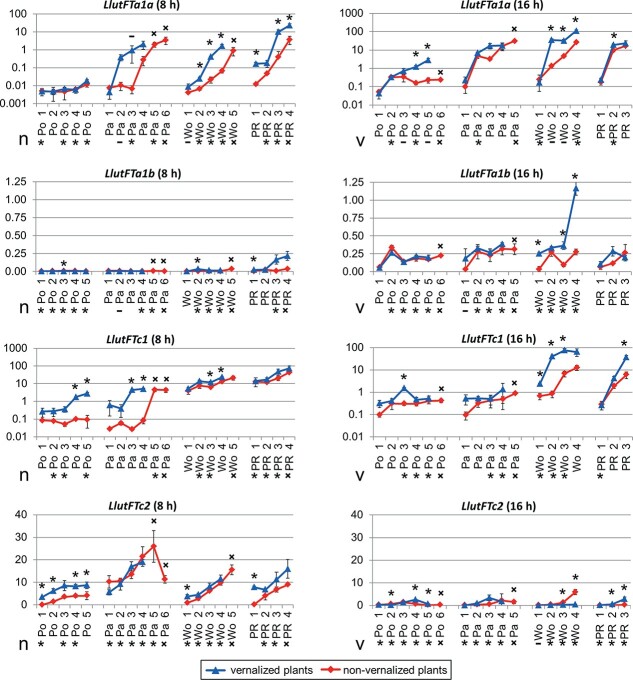
Expression profiles of *LlutFTa1a*, *LlutFTa1b*, *LlutFTc1* and *LlutFTc2* genes in yellow lupin accessions P28213 (Po), Parys (Pa), Wodjil (Wo) and PRH444/14 (PR). Numbers following accession abbreviations stands for sampling terms (Table S12). Three biological and three technical replicates were analyzed. Error bars show standard deviation. Two reference genes, a DEAD box RNA helicase 1 (*LlutDRH1*) and a beta tubulin 7 (*LlutTUB7*) were used for ∆∆Cq normalization. *LlutFTa1a* and *LlutFTc1* graphs are shown in log scale whereas *LlutFTa1b* and *LlutFTc2* in linear scale. Significance of vernalization influence on gene expression is shown above the data points. Significance of photoperiod influence is presented below the x axis (on the left panels for non-vernalized plants, n, and on the right panels for vernalized plants, v). ^*^, significant (*p* ≤ 0.05); no symbol, not significant; −, not calculated due to very different variance between groups, ×, not calculated due to the lack of corresponding data point for pairwise comparison.

### *LlutFTc1* and *LlutFTc2* genes co-localize with two major QTLs for yellow lupin flowering time

Based on the identified polymorphisms, 3 indel and 13 CAPS markers were developed for *LluFTa1a*, two CAPS markers for *LluFTa1b*, and single indel markers for the *LlutFTc1* and *LlutFTc2* (Table S8). Expected indel or and restriction enzyme cleavage products in parental lines were obtained for all markers. However, screening of the RIL mapping population revealed that two *LlutFTa1a* markers (FTa1a_F5_R5 and FTa1a_F13_R13) and one *LlutFTa1b* marker (FTa1b_M1_CAPS) were monomorphic. Moreover, segregation was significantly distorted (χ2 *p*-value 1E-11) from the expected 1:1 ratio for the remaining *LlutFTa1* markers. The *LlutFTc1* indel marker was localized in the linkage group YL-21 (2.9 cM, LOD values to surrounding markers 26.5 and 23.1), the *LlutFTc2* marker in the linkage group YL-01 (40.8 cM, LOD values 25.9 and 23.1), whereas the *LlutFTa1b* marker (FTa1b_F19_R20) at the end of the linkage group YL-60 (5.1 cM, LOD value 16.9). Markers with distorted segregation remained unmapped. QTL mapping was performed using linkage map updated with these markers and published data on flowering time in yellow lupin RIL population [31, 35]. Six statistically significant QTLs (1000 permutation test *p*-value <0.05) were identified (Table S9). These QTLs are localized on the linkage groups YL-01 (within the LOD_max-1_ range of 39.5–42.3 cM), YL-03 (20.9–25.7 cM), YL-06 (39.0–46.6 cM), YL-17 (38.0–41.0 cM), YL-21 (0.0–5.0 cM) and YL-34 (3.6–8.9 cM). *LlutFTc1* and *LlutFTc2* markers were found to be localized directly in the major QTL peaks (Fig. 4), explaining approximately 11% (*LlutFTc1*) and 25% (*LlutFTc2*) of observed phenotypic variance (flowering time of non-vernalized plants). Moreover, *LlutFTc1* marker matched the key locus for vernalization responsiveness in yellow lupin RIL population.

As a finished genome assembly is not available for yellow lupin, synteny with the better-characterized close relative, narrow-leafed lupin, was explored. All yellow lupin flowering time QTLs revealed patterns of shared collinearity (Table S10). The majority of these blocks carried known regulators from vernalization and photoperiod pathways. Thus, the QTL on linkage group YL-01 was in a collinear region of NLL-17 carrying the *LanFTc2* gene and the QTL on YL-21 was in a collinear region of NLL-10 encoding the *LanFTc1* gene. Moreover, the QTL on YL-03 matched the NLL-02 region containing the *LanCOL-9* gene and the QTL on YL-06 was syntenic to two NLL-20 regions separated by a break of collinearity; one of these regions carries the *LanFTa1* gene (Table S10).

**Table 4:** Candidate transcription factors identified for the *LlutFTa1a*, *LlutFTc1* and *LlutFTc2* genes

**Gene**	**Line**	**Locustype**	**transcription factor name**	**Accession**	**Position**	**Score**	**No. of other sites** ^**a**^	***FT*promoterbinding** ^**b**^	**Effect on flowering time** ^**b**^
*LlutFTa1a*	Wodjil	SNP	*BZIP52*	AT1G06850	2163	0.90	0		
	Wodjil	SNP	*GRF6*	AT2G06200	652	1.00	1		
	P28213	SNP	*TOE2*	AT5G60120	2328	0.93	0	+	+
*LlutFTc1*	P28213	Indel1	*AGL15*	AT5G13790	4387	1.00	0	+	+
	P28213	Indel1	*AGL15*	AT5G13790	5591	0.96	0	+	+
	P28213	Indel1	*AGL71*	AT5G51870	4511	0.84	0		−
	P28213	Indel1	*SUF4*	AT1G30970	3870	0.73	0		+
	P28213	Indel1	*TCP23*	AT1G35560	5781	0.92	2		+
	P28213	Indel1	*VRN1*	AT3G18990	5105	0.90	0	+	−
	P28213	Indel1	*VRN1*	AT3G18990	5548	0.92	0	+	−
*LlutFTc2*	P28213	SNP	*MYB62*	AT1G68320	6686	0.93	2		+

a the number of binding sites found for this transcription factor in the monomorphic regions in the promoter

b information based on the literature data [43–53] (see Discussion). Blanks indicate no reported effect on flowering time.

### *FT* genes and alleles differ in responsiveness to vernalization and photoperiod

Four yellow lupin lines differing in time to flowering and vernalization responsiveness (PRH444/14, Wodjil, Parys and P28213) were subjected to *LlutFTa1a*, *LlutFTa1b*, *LlutFTc1* and *LlutFTc2* gene expression profiling under two contrasting photoperiods (Tables S11 and S12, Fig. 5). Comparing mean values from all data points, *LlutFTa1a*, *LlutFTc1* and *LlutFTc2* genes revealed approximately 40–70 times higher expression levels than the *LlutFTa1b* gene (6.2 ± 15.4, 9.0 ± 16.8 and 4.9 ± 5.8 vs 0.13 ± 0.17, respectively). *LlutFTa1a*, *LlutFTc1* and *LlutFTc2* were considered as good candidate genes due to revealed association of sequence polymorphism with phenology traits and vernalization responsiveness, therefore their expression profiles are described herein in this context. Comparative analysis of expression levels between genotypes, photoperiod, vernalization variants, and growth phases is provided in Table S13. Moreover, *LlutFTa1a*, *LlutFTc1* and *LlutFTc2* genes revealed strong association between gene expression and timing of transition from vegetative to generative phases (Fig. 5).

Differences in expression profiles between genotypes were significant. In the absence of vernalization (Fig. 5), *LlutFTa1a* gene expression in Wodjil was higher than in P28213 up to 79-fold under SD (short day photoperiod) and up to 168-fold under LD (long day photoperiod). Similar observation was made for the *LlutFTc1* gene, reaching differences up to 225-fold and up to 42-fold, respectively. Moreover, *LlutFTa1a* expression in PRH444/14 was also higher than in P28213 (up to ~600-fold under SD and up to 48-fold under LD). A similar phenomenon was observed for the *LlutFTc1* gene in these lines (up to 423-fold difference under SD and up to 20-fold under LD). *LlutFTc2* expression pattern was more complex, putatively due to different photoperiod control. Taking into consideration photoperiod responses, *LlutFTc2* gene expression was negatively responsive to LD in all line × vernalization variants except the early dates in non-vernalized P28213 whereas the *LlutFTa1a* gene was highly induced by LD as compared to SD in all line × vernalization variants except the vernalized PRH444/14. *LlutFTc1* gene revealed significant LD induction in non-vernalized P28213 and vernalized Wodjil, significant LD repression in PRH444/14 and non-vernalized Wodjil as well as variable effects in other line × vernalization variants. Vernalization influence was significant. Thus, *LlutFTa1a* gene was positively responsive to vernalization under SD in Parys (up to 138-fold increase), PRH444/14 (up to 26-fold) and Wodjil (up to 25-fold), whereas under LD it was positively responsive in Wodjil (up to 26-fold), P28213 (up to 12-fold), and Parys (up to 5-fold). *LlutFTc1* gene was induced by vernalization under SD in Parys (up to 167-fold) and P28213 (up to 30-fold), whereas under LD in all studied lines (up to 47-fold in Wodjil, 6-fold in PRH444/14, and 5-fold in P28213 and Parys). *LlutFTc2* gene was induced by vernalization under SD in P28213 (up to 76-fold), PRH444/14 (up to 42-fold) and Wodjil (up to 4-fold), however, usually only in the first sampling date. Under LD this gene revealed repression by vernalization in Wodjil, induction in PRH444/14 and variable responses in P28213 and Parys.

### *FT* indels carry hypothetical binding sites of transcription factors from vernalization and photoperiod pathways

Promoter regions of *LlutFTa1a*, *LlutFTc1* and *LlutFTc2* genes were annotated for the presence of hypothetical binding sites of transcription factors (Table S14). The number of motif hits differing in the whole promoter sequences between P28213 and Wodjil reached 110–2260 for the *LlutFTc1* gene, 116–242 for the *LlutFTc2* gene and 92–218 for the *LlutFTa1a* gene. Analysis of polymorphisms found in the diversity panel revealed the presence of 11–228 motif hits for the *LlutFTa1a* gene and 6–704 motif hits for the *LlutFTc1* gene. Variability of transcription factor binding sites was highly associated with the presence of large indels, i.e. 2231 hits for *LlutFTc1* indel1, 693 hits for *LlutFTc1* indel4, 349 hits for *LlutFTc1* indel3, 215 hits for *LlutFTa1a* indel4. As the vast majority binding sites for particular transcription factors were present both in the polymorphic and monomorphic regions, the number of motifs found only in the polymorphic loci was much lower, reaching from 0 to 137 hits (Table 4).

As one polymorphic locus typically provided redundant hits, therefore the real number of candidate unique transcription factors even lower. Thus, just a few unique transcription factors which could participate in regulation of flowering time in response to photoperiod and vernalization were identified. Taking into consideration the reports from other studies providing evidence for *FT* promoter binding and/or control of flowering time (see Discussion), a narrow list of candidate transcription factor*s* was selected, including *TARGET OF EAT2* (*TOE2*) for the *LlutFTa1a* gene, *AGAMOUS*-like *15* (*AGL15*) for the *LlutFTc1* gene and *MYB62* for the *LlutFTc2* gene. (Table 4).

**Figure 6:**
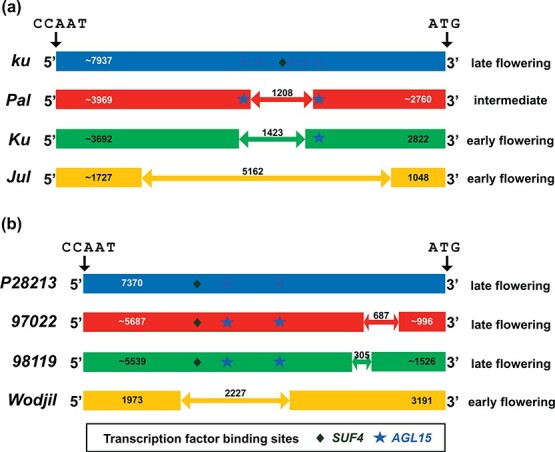
Comparison of indel variation in the promoter sequences of *LanFTc1* (a) and *LlutFTc1* (b) genes controlling flowering induction in *Lupinus angustifolius* and *L. luteus*, respectively. Position is given in base pairs in relation to the first nucleotide of CCAAT-box [34, 61]. *Ku*, *Jul*, *Pal* and *ku* are *LanFTc1* alleles, whereas P28213, 97 022, 98 119 and Wodjil represent *LluFTc1* alleles. Binding sites for transcription factors, AGAMOUS-like 15 (AGL15) and SUPPRESSOR OF FRI 4 (SUF4) were identified by in silico analysis using the Plant Promoter Analysis Navigator 3.0 [69].

As lengths and positions of major *LanFTc1* and *LlutFTc1* promoter indels are similar [34] it would be interesting to know if the sets of indel-specific transcription factor binding sites are also similar. Therefore, we analyzed *LanFTc1* indels in the same way as the *LlutFTc1* indels (Table S15). This analysis highlighted AGL15 as a candidate diversifying transcription factor for *LanFTc1* gene (Fig. 6), revealing 3 binding sites in *Pal* indel, 4 sites in *Ku* indel, 5 sites in *Jul* indel, and one site in the monomorphic region with much lower similarity score (0.81–0.82) than the sites in the indels (0.96–1.00). Moreover, in the *LanFTc1* indels, binding sites were found for AGL71 (two in the *Ku*, *Pal* and *Jul* indels, another two in monomorphic regions) and SUF4 (1 in *Ku*, *Pal* and *Jul*, 0 in monomorphic regions). No candidate binding site for VRN1 was found in the whole *LanFTc1* promoter.

## Discussion

### Yellow lupin duplicates of *FTa* and *FTc* homologues as remnants of a lineage-specific ploidy event.

The number of *FTa* and *FTc* homologues revealed in yellow lupin genome in the present study is the same as in the narrow-leafed and white lupin genomes [18, 39, 40]. Bayesian inference provided evidence for split into *FTa*, *FTb* and *FTc* clades before the divergence of major Papilionoideae lineages [41]. This observation supports the concept that a simultaneous divergence of all major legume subfamilies was associated with mass extinction at the Cretaceous–Paleogene boundary (66 million years ago) and a whole-genome duplication event [54–56]. Additional polyploidy events occurred later in downstream lineages, including lupin (hypothesized triplication) and soybean (duplication) [42, 57, 58]. Remnants of these processes can still be found in plant genomes in the form of additional gene copies arranged in collinear blocks. The mechanisms conferring the retention of duplicated genes are not well understood, nevertheless, it was revealed that high retention rates, include, among others, genes from flowering and cold-responsive pathways [59, 60]. The presence of particular lupin *FTa* and *FTc* paralogs in monophyletic clades (Fig. 1) suggests their origin by duplication in the ancestral *Lupinus* lineage. Interestingly, lupin species lost the whole *FTb* clade, which is very abundant in other legumes, whereas they retained duplicates of the *FTc* which is usually a single copy clade [41].

### Sub-functionalization of *FTc1* into vernalization and *FTa1* into photoperiod in lupins

The present study revealed sub-functionalization of *LlutFTc1* into vernalization pathway (Fig. 2–4), whereas *LlutFTa1a* into photoperiod response (Fig. 5). A similar observation was made for *LanFTc1* (wild allele) and *LanFTa1* (Palestinian allele) genes in narrow-leafed lupin [18, 61]. Unfortunately, providing direct evidence for these functions by targeted reverse genetics is currently impractical due to the constraints of the lupin transformation system. In other legume species, *FT* duplicates also revealed functional divergence between photoperiod and vernalization pathways, such as genes *MtFTb1* and *MtFTb2* vs *MtFTa2* in *Medicago truncatula* or genes *PsFTb2* vs *PsFTa1* in *Pisum sativum*, respectively [12, 14, 62]. Incorporation of different *FT* homologs into vernalization pathway in legumes is not surprising when the timeline of ancient climate changes is placed in the evolutionary context. Vernalization as a trait likely evolved in a response to major global cooling that peaked during the Eocene–Oligocene boundary 34 million years ago, as evidenced for temperate Pooideae grasses [63, 64]. Therefore, a general mechanism of vernalization response based on the *FTc* clade may have been established several million years before the ploidy event in the *Lupinus* lineage [58, 65]. Following duplication, *FTc1* orthologs retained basic functions whereas *FTc2* differentiated in downstream lineages, resulting in the loss-of-function in narrow-leafed lupin [18, 61] and partial sub-functionalization in yellow lupin (Fig. 2-5). The other evidence supporting relatively recent evolution of vernalization trait is the observed lack of conservation of *Arabidopsis FRIGIDA*-*FLC* model in many species, including the above-mentioned Pooideae grasses [64, 66]. The same phenomenon can be expected in legumes, as many of them, including lupins, did not retain any copy of the major integratory gene from the vernalization pathway, *FLC* found in the Brassicaceae [65, 67].

### AGL15 as a candidate *FTc1* transcription factor controlling vernalization requirement and vegetative phase duration

The present study evidenced the association between indel polymorphism in regulatory region of *LlutFTc1* and vernalization-independent flowering (Fig. 2). A similar observation was reported for narrow-leafed lupin and the series of *LanFTc1* indels [34]. In *Arabidopsis*, the *FT* promoter region is relatively long (~5 kbp) and carries numerous binding sites for regulatory agents from photoperiod, light quality, vernalization and aging pathways [43, 68]. Our study (Table 4) revealed that four transcription factors have specific candidate binding sites in the polymorphic regions of the *LlutFTc1* promoter: AGAMOUS-like 15 (AGL15), AGL71, SUPPRESSOR OF FRI 4 (SUF4) and VERNALIZATION 1 (VRN1). Comparative analysis of candidate binding sites in *LlutFTc1* and *LanFTc1* promoters designated AGL15 as a candidate transcription factor diversifying between particular structural variants (Fig. 6).

AGL15 is a MADS-box transcription factor that acts as floral repressor during vegetative phase by binding *FT* promoter sequence at sites that partially overlap those bound by FLC and SHORT VEGETATIVE PHASE (SVP) proteins [43]. The *LlutFTc1* indel carries all candidate AGL15 binding sites found in the whole promoter (Table 4). Therefore, hypothetical repression of the *LlutFTc1* gene by AGL15 may occur in the late flowering Parys and P28213 lines whilst it should not occur in the early flowering lines lacking appropriate binding sites. High expression of *LlutFTc1* observed in PRH444/14 and Wodjil lines beginning with the juvenile phase highlights AGL15 as a major candidate for the *LlutFTc1* indel-related early flowering. A similar conclusion can be made for the *LanFTc1* (narrow-leafed lupin) indels.

The second candidate supported by in silico indel analysis in both lupin species, SUF4, controls vernalization dependence by binding *FLC* chromatin as a component of FRIGIDA transcription activator complex [44]. Nevertheless, to our knowledge, there is no evidence for SUF4 to bind the *FT* promoter. Moreover, in *Arabidopsis*, SUF4 activates transcription of the target, whereas in our study candidate binding site was found in the wild allele, indicating expected repressive activity. The third candidate, supported by indel analysis only in yellow lupin, VRN1, is a B3 domain carrying transcription factor associated with *Arabidopsis* flowering in response to vernalization [45]. VRN1 constitutes a hypothetically eudicot-specific component of PRC1-like complex, which is one of the two Polycomb complexes involved in epigenetic silencing of an *FLC* gene [46, 47]. PCR1-like activity is also linked with epigenetic control of *FT*, enabling temperature-responsive flowering time regulation [70]. Due to presence of VRN1 binding sites only in the wild allele, such vernalization-driven silencing of *LlutFTc1* should lead to opposite expression and flowering time profiles than observed, therefore this mechanism is unlikely. The last candidate, AGL71, is a MADS-box transcription factor acting downstream of *SOC1* and promoting flowering in the shoot apical and axillary meristems under the gibberellin-dependent pathway [48]. However, we disregarded this transcription factor due to presence of additional candidate binding sites also in the monomorphic region of the *LanFTc1* promoter.

### Disruption of enhancer chromatin loop formation by *FTc1* promoter indels is unlikely

The other possible mechanism that could explain the observed difference in phenotypes associated with *LlutFTc1* promoter variants is related with protein-mediated interaction between structural components of the *FT* promoter [71]. Two such motifs, CCAAT and RE-alpha, were also found in narrow-leafed lupin *FT* promoters at conserved positions [41]. In *Arabidopsis*, CCAAT sequences serve as binding sites for the NUCLEAR FACTOR Y (NF-Y) – CONSTANS (CO) complex facilitating formation of long-distance chromatin loop bringing distal enhancer elements into close association with the proximal CO-responsive elements (CORE1 and CORE2) [72–74]. The functional consequence of this interaction is reduction of PcG protein levels at the *FT* promoter, relieving this region from Polycomb silencing under inductive photoperiod [75]. A large *LlutFTc1* indel reported in this study carries six CCAAT elements (Table S14), whereas *LanFTc1* indel variants carry from one to several such motifs [41]. Nevertheless, in both species there were additional CCAAT elements identified, flanking these indels, which may eventually participate in chromatin loop formation. Moreover, eventual disruption of chromatin looping at *LlutFTc1* or *LanFTc1* promoters by indels should result in the opposite phenotypic effects than observed.

### TOE2 as a candidate *LlutFTa1a* transcription factor controlling photoperiod response

The present study identified three candidate transcription factors (Table 4) for the *LlutFTa1a* gene: BASIC LEUCINE ZIPPER 52 (bZIP52), GROWTH REGULATING FACTOR 6 (GRF6) and TARGET OF EAT 2 (TOE2). In *Arabidopsis*, bZIP52 protein is involved in heat stress response [49]. GRF6, known as a 14–3-3 protein, induces rice flowering by interaction in shoot apical meristem with FT and FLOWERING LOCUS D (FD) proteins to activate the floral promoter *SUPPRESSOR OF OVEREXPRESSION OF CO 1* (*SOC1*) and downstream floral meristem identity genes [50]. However, no evidence for binding *FT* promoter by GRF6 was found in literature data. The latter transcription factor, TOE2, is a component of photoperiodic pathway and represses *FT* transcription by binding to its chromatin, preventing flowering under short days [51, 52]. The presence of a TOE2 candidate binding site only in the wild (P28213) *LlutFTa1a* promoter allele supports the hypothesis on the involvement of TOE2 in photoperiod-related flowering control in yellow lupin.

### *Copia-like* retrotransposon insertion at the *LlutFTc2* gene may delay flowering under non-inductive photoperiod

A large (5269 bp) insertion of a *Copia-like* retrotransposon in the third intron of the *LlutFTc2* was associated with delayed flowering (Fig. 2 and 4). Moreover, *LlutFTc2* gene expression in P28213 line carrying this insertion was significantly reduced under short days (Fig. 5). A similar phenomenon was observed in soybean, where insertion of a *Copia-like* element (6224 bp) in the first intron of the soybean *FT* homologue (*GmFT2a*) was associated with decreased expression of this gene and delayed flowering [17]. A Tgm-like transposon insertion in the third intron of the *GmFT2c* gene that occurred at the early stage of soybean domestication also caused later flowering than the wild allele [76] Similarly, insertion of the *Tnt1* retrotransposon within the first intron of the *M. truncatula FT* homologue (*MtFTa1*) resulted in a late-flowering phenotype [12]. In *Arabidopsis*, insertion of the Mutator-like transposable element in the first intron of the *FLC* gene conferred early flowering phenotype based on epigenetic silencing of *FLC*, mediated by short interfering RNAs [77]. Thus, the first intron of the *FLC* gene is relatively long and it is frequently targeted by transposons and retrotransposons in Brassicaceae species, providing significant transcriptional and phenotypic variation [78]. In *A. thaliana* most long introns are enriched with heterochromatic transposable element sequences [79]. Interestingly, the third introns of *FT* genes in three lupin species with sequenced genomes (*L. angustifolius*, *L. albus* and *L. luteus*) are also relatively long (from about 1.5 kbp to 6.5 kbp), therefore a similar mechanism like in *Arabidopsis FLC* may be expected. Apart from commonly observed deleterious effects, insertion of transposable elements can provide adaptive variation and facilitate evolutionary response to rapid environmental changes [80].

### *FT* indel polymorphism provides high flexibility in modification of yellow lupin phenology by traditional breeding

In this study, *LlutFTc1* indel allele conferring vernalization independence was found only in domesticated yellow lupin germplasm except one landrace from Palestine. A similar scenario was revealed for *LanFTc1* indels in narrow-leafed lupin [34, 61]. To our knowledge it is the first example of such a convergence of *FT* indel evolution addressing artificial selection during domestication process and adaptation to environmental conditions that favors short season (Palestinian allele) between two related species. Both species (yellow lupin and narrow-leafed lupin) had lost vernalization requirement through large deletions in the promoter regions of the same *FTc1* homologue, a central integrator of flowering time. This provides a unique opportunity to explore the molecular regulation of flowering time in these related species. It also provides motivation to prospect for additional examples of *FTc1* deletions in other lupin species. This study provides for the first time a molecular marker that is perfectly predictive of vernalization responsiveness in yellow lupin. This can be used to facilitate introgression of new genetic diversity into domesticated germplasm (without vernalization requirement) from wild types (primarily with vernalization requirement). Moreover, the yellow lupin diversity panel offer very high flexibility for selection for vernalization and photoperiod responsiveness/independence due to the presence of lines carrying different allelic combinations of *LlutFTa1a*, *LlutFTc1* and *LlutFTc2* indels. High variability in allelic composition resulted in large phenotypic variance of flowering time and vernalization responsiveness, including numerous intermediate phenotypes with upgraded domestication status awaiting further exploitation by classic breeding.

## Materials and methods

### Yellow lupin germplasm material

A panel of yellow lupin accessions was assembled representing the full spectrum of diversity for this species with seed provided by the Poznań Plant Breeders Ltd. (Wiatrowo, Poland) and Plant Breeding Smolice Ltd. (Przebędowo, Poland). The panel (Table S1) comprised 111 accessions (3 wild types, 5 landraces, 4 mutants, 33 cross-derivatives / breeding lines and 66 cultivars). A mapping population of 97 recombinant inbred lines (RILs) along with parental controls (P28213 and Wodjil) was provided by the Department for Primary Industries and Regional Development (South Perth, Australia).

### Phenotyping of yellow lupin phenology and vernalization responsiveness

Vernalization was performed by placing seeds for 21 days at 5°C on moist filter paper in Petri dishes in darkness. Non-vernalized control plants were sown four days before the end of vernalization treatment and grown at 24°C to maintain similar thermal time. Plants were cultivated in a greenhouse located at the Institute of Plant Genetics, Polish Academy of Sciences, Poznań, Poland (52°26’N 16°54′E) during the growing seasons of 2016 (sowing date 23.03), 2017 (sowing date 27.03) and 2019 (sowing date 25.03) under ambient long day photoperiods (~12–17 h). Phenology observations included bud emergence (counted as days from sowing to the first bud appearance), start of flowering (recorded when the first fully colored petal was observed) and end of flowering (recorded when most of petals on the main stem faded). The number of observed replicates varied between 3 and 10 (mean value of 6.2) depending on germination rate and plant survival during the experiments.

### Calculation of heritability and interactions

The linear mixed-effect model was used to estimate variance components and predict the genetic values via single-trait BLUP (best linear unbiased prediction). A lmer function was used to fit the model [81] from lmer4 (version 1.1–29) R 4.1.0 package [82]. Using variance components, the phenotypic variance, the broad-sense heritability, the heritability on the mean basis, the selective accuracy (the correlation between the predicted and true genotypic values), a genotype-environment correlation, a genotypic coefficient of variation and a residual coefficient of variation were calculated [83]. The vernalization effect on flowering time in investigated genotypes was tested using the estimated marginal means method [84]. Using the emmeans function from emmeans (version 1.5.4) R package, a combination of effect of genotypes and vernalization from a linear mixed-effect model was used in pairwise comparison. As a multiplicity adjustment method “tukey” was applied.

### Sequencing of the yellow lupin *FT* homologues

Coding sequences of *L. angustifolius FT* homologs, *LanFTc1*, *LanFTc2*, *LanFTa1a* and *LanFTa1b* [41] were aligned to the yellow lupin genome scaffolds (N = 2458, N50 = 1.5 Mbp, unpublished) using progressive Mauve algorithm with gapped aligner MUSCLE 3.6 [85, 86] implemented in Geneious v8.1 [87]. Gene features in selected scaffolds were annotated in FGENESH+ [88] using the *Glycine max* model and *L. angustifolius* FT protein sequences as references. The nucleotide sequences of *FT* homologues were analyzed in four yellow lupin accessions differing in flowering time and vernalization responsiveness (PRH444/14, Wodjil, Parys and P28213). Young leaves were collected from 5-week-old plants cultivated in a greenhouse. DNA was isolated using DNeasy Plant Mini Kit (Qiagen). Based on *FT* sequence annotations, a series of overlapping PCR primer pairs covering the entire gene sequences from ~8 kbp promoter to 3′ untranslated regions were designed (Table S4). The lengths of targeted genomic regions were 12 197 bp for the *LlutFTa1a* gene, 11 103 bp for the *LlutFTa1b*, 15 260 bp for the *LlutFTc1* and 17 236 bp for the *LlutFTc2*. Standard sized (up to 2 kbp) PCR products were amplified using GoTaq G2 Flexi DNA Polymerase (Promega, Mannheim, Germany) whereas longer products used GoTaq® Long PCR Master Mix (Promega). Amplicons were directly Sanger-sequenced using BigDye® Terminator v3.1 Cycle Sequencing Kit (Applied Biosystems) and 96-capillary 3730xl DNA Analyzer (Applied Biosystems) by Genomed (Warsaw, Poland). Final *FT* sequences were assembled using *de novo* assembler in Geneious and aligned to each other using a progressive Mauve algorithm. Bayesian inference of *FT* coding sequences [12, 14, 41, 42] was performed as previously described [41].

### Linkage mapping of *FT* genes and flowering time QTL loci

Molecular markers anchored in *FT* polymorphisms were designed to localize *FT* homologues on the yellow lupin linkage map [31] (Table S8). Standard agarose gel electrophoresis was used for visualization of indel markers whereas Cleaved Amplified Polymorphic Sequence (CAPS) approach [89] for single nucleotide polymorphisms (SNPs). Restriction sites and corresponding enzymes were identified using dCAPS Finder 2.0 [90]. Restriction enzymes were supplied by Thermo Fisher Scientific (Warsaw, Poland) and New England Biolabs (Ipswich, USA). Chi-square (χ2) values for Mendelian segregation in F_8_ RILs were estimated using the expected 1:1 segregation ratio (disregarding heterozygotes). Published marker segregation data [31] and those developed in this study were imported to Map Manager QTXb20 [91] and distributed under *p*-value of 0.001 to the positions at which their insertions caused the greatest increase in the sum of LOD linkage scores for adjacent loci. Kosambi function was used to calculate map distances. Data on flowering time [31] and the updated linkage map from this study were exploited for composite interval mapping using Windows QTL Cartographer V2.5 (window size 10 cM and walk speed 0.5 cM). To test the stability of identified QTLs, calculations were performed within the range from 1 to 10 background control markers. Using the same parameters, permutation tests (×1000) were performed to establish LOD thresholds. Linkage groups were drawn using MapChart [92].

### Correlation between genotype (*FT* indel polymorphism) and phenotype (phenological traits)

To survey distribution of *FT* indel polymorphism and find eventual novel variants in lines with contrasting phenology, yellow lupin diversity panel was screened by PCR and agarose gel electrophoresis with the same primers as those used for *FT* sequencing (Table S4). Putatively novel indel alleles were confirmed by Sanger sequencing. Reference alleles (Wodjil) were coded as 1, alternative alleles as 2, additional alternative alleles (the least frequent) as 3, whereas heterozygotes as 1.5 (presence of alleles 1 and 2) or 2.5 (presence of alleles 2 and 3). Association between genotype and phenotype (flowering time and vernalization response) was calculated as Spearman’s rank correlation between alleles and BLUP values. To check if the revealed associations could be considered as statistically significant by normal standards, *p*-value was calculated using cor.test R base function. Correlation values were visualized using heatmap function from Complexheatmap (version 1.10.2) R package [93]. Promoter regions of FT genes were annotated for hypothetical transcription factor binding sites using the Plant Promoter Analysis Navigator 3.0 [69].

### Expression profiling of yellow lupin *FT* genes in response to photoperiod and vernalization

Vernalization and sowing procedures were as described above. Plants were cultivated in climatic chambers with controlled humidity (40–50% day, 70–80% night) and temperature (22°C day, 18°C night). Two levels of photoperiod were applied, short day (SD, 8 h) and long day (LD, 16 h). Young leaves were sampled every week one hour before the end of the light phase, covering the period from about 2–3 weeks before floral bud emergence until flowering (Table S12). Plant material was immediately frozen in liquid nitrogen and stored at −80°C. SV Total RNA Isolation System (Promega) was used for RNA isolation. Concentration and quality were measured using a NanoDrop 2000 (ThermoFisher Scientific). Additional quality control was performed for 60 isolates using Experion™ Automated Electrophoresis System and Experion RNA StdSens Analysis Kit (Bio-Rad, Hercules, CA, USA). The first-strand cDNA synthesis was performed using iScript cDNA Synthesis Kit (Bio-Rad) and 1 μg of total RNA per sample. The set of analyzed genes (Table S16) included *LlutFTa1a*, *LlutFTa1b*, *LlutFTc1* and *LlutFTc2* genes) and two references – a homolog of DEAD box RNA helicase 1 (*LlutDRH1*) and beta tubulin gene (*LlutTUB7*). Gene expression profiling was performed using a CFX Connect Real-Time PCR Detection System (Bio-Rad). Standard curves were developed following previously reported protocol [61]. R [2] and PCR efficiency values (Table S16) were calculated using Bio-Rad CFX Manager 3.1. Three biological replicates (each with three technical replicates) including inter-run calibration samples (*LlutTUB7*) and no-template controls were analyzed. High resolution DNA melting was performed after PCR to control the specificity of amplification. Calculations of ∆∆Cq included both reference genes. Effects of growth phase (expression at analyzed date divided by expression at the first date), vernalization (x-fold change of expression after vernalization), photoperiod (x-fold change of expression of SD versus LD or vice versa) and genotype were analyzed. Statistical significance was tested using t test for mean ratio [94,95]. Calculations were made in R with custom script using “t.test.ratio” function from the mratios (version 1.4.2) package. First, the equal of variance was tested; if this condition was satisficed the classical t-test was used; otherwise, the Welch’s t-test formula was used [96]. If variances were significantly different (p-value <0.001) it was assumed that the results come from different populations and calculation was not performed [97]. To evaluate stability of reference genes during vernalization, mean efficiency-corrected Cq values obtained for reference genes were compared between vernalized and non-vernalized accessions revealing non-significant differences for all studied lines (Table S17).

## Acknowledgements

Authors would like to thank Joshua A. Udall from Southern Plains Agricultural Research Center, Agricultural Research Service, U.S. Department of Agriculture, College Station, TX, USA for the prepublication access to the yellow lupin genome assembly. Moreover, authors acknowledge Magdalena Tomaszewska from Legume Genomics Team of the Institute of Plant Genetics, Polish Academy of Sciences for help in plant sowing and performing phenotypic observations in greenhouse. This research was funded by the Ministry of Agriculture and Rural Development, Poland (Basic Research for Biological Progress in Crop Production, task 39) and National Science Centre, Poland (OPUS21, 2021/41/B/NZ9/02226). An open access charge was co-financed by Wroclaw University of Environmental and Life Sciences as well as by National Science Centre, Poland.

## Contributions

MK designed the experiments. PP, SR, MMI and IJM-B performed the research and data collection. PP, SR-B, MK, BK performed data analysis and interpretation. MK and MNN wrote the manuscript.

## Data availability statement

Annotated *LlutFTa1a*, *LlutFTa1b*, *LlutFTc1* and *LlutFTc2* gene sequences with promoter regions assembled for Wodjil, PRH444/14, Parys and P28213 lines as well as PCR product sequences representing other *FT* indel variants found in diversity panel and regions of reference genes used in quantitative PCR were deposited in the DNA Data Bank of Japan under accession numbers LC663825-LC663836, LC664023-LC664026, LC664167-LC664174, LC666892-LC666899. Custom R scripts were deposited in GitHub repository (https://github.com/igrlupin/Yellow-lupin-paper).

## Conflicts of interest

The authors declare no conflict of interest. The funders had no role in the design of the study; in the collection, analyses, or interpretation of data; in the writing of the manuscript, or in the decision to publish the results.

## Supplementary data

Supplementary data is available at *Horticulture Research * online.

## Supplementary Material

supp_data_uhac180
